# The Effects of Tempol on Cyclophosphamide-Induced Oxidative Stress in Rat Micturition Reflexes

**DOI:** 10.1155/2015/545048

**Published:** 2015-04-20

**Authors:** Eric J. Gonzalez, Abbey Peterson, Susan Malley, Mitchel Daniel, Daniel Lambert, Michael Kosofsky, Margaret A. Vizzard

**Affiliations:** Department of Neurological Sciences, University of Vermont College of Medicine, Burlington, VT 05405, USA

## Abstract

We hypothesized that cyclophosphamide- (CYP-) induced cystitis results in oxidative stress and contributes to urinary bladder dysfunction. We determined (1) the expression of oxidative stress markers 3-nitrotyrosine (3-NT), reactive oxygen species (ROS)/reactive nitrogen species (RNS), inflammatory modulators, neuropeptides calcitonin gene-related peptide (CGRP), substance P (Sub P), and adenosine triphosphate (ATP) that contribute to the inflammatory process in the urinary tract and (2) the functional role of oxidative stress in urinary bladder dysfunction with an antioxidant, Tempol, (1 mM in drinking water) combined with conscious cystometry. In CYP-treated (4 hr or 48 hr; 150 mg/kg, i.p.) rats, ROS/RNS and 3-NT significantly (*P* ≤ 0.01) increased in urinary bladder. CYP treatment increased ATP, Sub P, and CGRP expression in the urinary bladder and cystometric fluid. In CYP-treated rats, Tempol significantly (*P* ≤ 0.01) increased bladder capacity and reduced voiding frequency compared to CYP-treated rats without Tempol. Tempol significantly (*P* ≤ 0.01) reduced ATP expression, 3-NT, and ROS/RNS expression in the urinary tract of CYP-treated rats. These studies demonstrate that reducing oxidative stress in CYP-induced cystitis improves urinary bladder function and reduces markers of oxidative stress and inflammation.

## 1. Introduction

Bladder pain syndrome (BPS)/interstitial cystitis (IC) is a chronic syndrome characterized by pressure, discomfort, and pain thought to arise from the urinary bladder, with at least one urinary symptom [1, 2]. While the underlying etiology of BPS/IC is not known, the majority of biopsies from BPS/IC patients reveal inflammation [3]. Mediators of inflammation, including cytokines, chemokines, growth factors, and neuropeptides, have been shown to contribute to urinary bladder dysfunction and somatic sensitivity in animal models of cystitis and in the clinical syndrome of BPS/IC [3, 4]. In addition, reactive oxygen species (ROS) and reactive nitrogen species (RNS) generated by inflammation result in oxidative stress and may contribute to urinary bladder dysfunction [5, 6]. The role(s) that oxidants may have in inducing inflammation has been extensively studied in diverse experimental models [7]. Although it is widely accepted that ROS/RNS are fundamentally involved, antioxidant therapy as a valid means of arresting inflammation remains largely unresolved especially in the context of urinary bladder inflammation.

Accompanying oxidative stress and inflammatory mediator upregulation, the urothelium may also respond to cystitis by increasing the secretion of neuroactive factors, such as ATP, which signal to the underlying nerve plexus [8]. Altered release of these neuromodulatory compounds has been suggested to contribute to increased sensory transduction and result in urinary bladder dysfunction [4, 8]. In BPS/IC, for example, distention-evoked release of ATP was increased in tissue biopsies that may have resulted in the elevated urinary ATP observed in patients with BPS/IC [9]. Similarly, numerous animal models of cystitis have demonstrated increased distention-evoked release of ATP from the urothelium [10, 11]. Further, purinergic receptor activation has been associated with increased cellular production and release of multiple inflammatory mediators, including superoxide anion, nitric oxide, and other ROS [12]. Purinergic receptor activation induces ROS generation in numerous cell types resulting in a variety of downstream effects including transcription factor activation [13], proinflammatory cytokine release [14, 15], and cell death [16].

Using a rat model of urinary bladder inflammation induced by cyclophosphamide (CYP) [3, 4, 17, 18], we determined (1) the expression of oxidative stress markers (3-nitrotyrosine (3-NT), ROS/RNS) and other modulators of inflammation, the neuropeptides calcitonin gene-related peptide (CGRP) and substance P (Sub P) in the urinary tract; (2) the contribution of ROS/RNS to ATP expression in the urinary bladder and urine with an antioxidant and superoxide dismutase mimetic, Tempol (1 mM in drinking water); and (3) the role of ROS/RNS in urinary bladder function with Tempol combined with open outlet and conscious and continuous intravesical infusion [19]. Although the etiology of BPS/IC is unknown, previous studies [3, 4, 17, 18] have demonstrated that the rat CYP model of urinary bladder inflammation is a reliable and reproducible model with face validity (e.g., increased voiding frequency and referred somatic sensitivity) to BPS/IC. Previous studies have demonstrated roles for neuropeptides, including CGRP and Sub P, in CYP-induced bladder dysfunction [20, 21] and the current studies have continued to focus on these two neuropeptides, abundantly expressed in bladder sensory pathways.

## 2. Materials and Methods

### 2.1. Animals

Adult female, Wistar rats (200–225 g; Charles River, St. Constant, Canada) were used for this study. Rats were housed two per cage and maintained in standard laboratory conditions with free access to food and water. The University of Vermont Institutional Animal Care and Use Committee approved all animal use procedures (protocol 08-085). Animal experimentation was carried out in accordance with the National Institutes of Health Guide for the Care and Use of Laboratory Animals. All efforts were made to minimize the potential for animal pain, stress or distress.

### 2.2. Induction of Cyclophosphamide- (CYP-) Induced Cystitis

Rats were anesthetized under isoflurane (2%) and acute cystitis was induced with a single injection of CYP (150 mg/kg, i.p.) and used in studies at various time points (4 hours (hr) or 48 hr) after treatment [22–24]. Control rats received volume-matched injections of saline (0.9%; i.p.) or no treatment and no difference among the control groups was observed. All injections were performed under isoflurane (2%) anesthesia. The CYP model of bladder inflammation produces an increase in voiding frequency with small micturition volumes and is associated with inflammatory cell infiltrates in the urinary bladder including mast cells, macrophages, and neutrophils [25, 26]. Rats were euthanized using isoflurane (5%) and a thoracotomy.

For cystometry in conscious rats, an unrestrained animal was placed in a Plexiglas cage with a wire bottom. Before the start of the recording, the bladder was emptied and the catheter was connected via a T-tube to a pressure transducer (Grass Model PT300, West Warwick, RI) and microinjection pump (Harvard Apparatus 22, South Natick, MA). A Small Animal Cystometry Lab Station (MED Associates, St. Albans, VT) was used for urodynamic measurements [19, 27]. Saline solution was infused at room temperature into the bladder at a rate of 10 mL/h to elicit repetitive bladder contractions. At least six reproducible micturition cycles were recorded after the initial stabilization period of 25–30 min [19, 27]. The following cystometric parameters were recorded in each animal: baseline pressure (BP; pressure at the beginning of the bladder filling), threshold pressure (TP; bladder pressure immediately prior to micturition), peak micturition pressure (MP), intercontraction interval (ICI; time between micturition events), bladder capacity (BC), void volume (VV), and presence and amplitude of nonvoiding bladder contractions (NVCs) [19, 27]. NVCs were defined as rhythmic intravesical pressure increases 7 cm H_2_O above baseline, during the filling phase, without the release of fluid from the urethra. Bladder capacity (BC) was measured as the volume of saline infused into the bladder at the time when micturition commenced [28]. In these rats, residual volume was less than 10 *μ*L; therefore, VV and BC were similar. After the initial stabilization period, cystometric fluid expelled during micturition events was collected and frozen on dry ice and stored at −80°C for future use in the assays described above. At the conclusion of the experiment, the rat was euthanized (5% isoflurane plus thoracotomy); the urinary bladder was harvested and used in the assays described above.

### 2.3. Split Bladder Preparation and Assessment of Potential Contamination of Bladder Layers

The urothelium + suburothelium was dissected from the detrusor smooth muscle using fine forceps under a dissecting microscope as previously described [22, 23]. To confirm the specificity of our split bladder preparations, urothelium + suburothelium and detrusor samples were examined for the presence of *α*-smooth muscle actin (1 : 1000; Abcam, Cambridge, MA) and uroplakin II (1 : 25; American Research Products, Belmont, MA) by western blotting or reverse transcription PCR [22, 29]. In urothelium + suburothelium layers, only uroplakin II was present (data not shown). Conversely, in detrusor samples, only *α*-smooth muscle actin was present (data not shown). All subsequent measurements of urinary bladder and cystometric fluid samples and conscious cystometry in rat groups were performed in a blind manner.

### 2.4. Substance P (Sub P), Calcitonin Gene-Related Peptide (CGRP), and 3-Nitrotyrosine (3-NT) by Enzyme-Linked Immunosorbent Assays (ELISAs)

Tissue processing and ELISAs were performed as described previously [18, 30]. Briefly, rats from control (*n* = 6 each) and all experimental groups (*n* = 6 each) were deeply anesthetized (4% isoflurane), and a thoracotomy was performed. Individual rat bladders were dissected, weighed, and placed in Tissue Protein Extraction Reagent (1 g tissue/20 mL; Pierce Biotechnology, Woburn, MA) with complete protease inhibitor cocktail tablets (Roche Applied Science, Mannheim, Germany) and stored at −80°C. On the day of assay, individual bladders were disrupted with a Polytron homogenizer until being homogeneous and centrifuged (10,000 rpm for 10 min) [18, 30], and the supernatant was used for total protein estimation and CGRP (Phoenix Pharmaceuticals, Inc., Burlingame, CA), Sub P (Phoenix Pharmaceuticals), and 3-NT (Millipore Corporation, Bellerica, MA) quantification. Total protein was determined by the Coomassie Plus Protein Assay Reagent Kit (Pierce) [18, 30] and CGRP, Sub P, and 3-NT were quantified using standard 96-well ELISA plates (Phoenix Pharmaceuticals; Millipore Corporation) according to the manufacturer's recommendations. For determination of Sub P and CGRP content in voided cystometric fluid, void volumes were collected following each micturition event in control and CYP-treated groups with and without Tempol (only vehicle) administration during conscious cystometry (see below). For Sub P, CGRP, and 3-NT measurements in voided cystometric fluid, 6–8 individual voids/animal were collected and immediately frozen on dry ice. Data from multiple voids were averaged, and the mean value was used for each animal.

### 2.5. ELISAs for CRGP, Sub P, and 3-NT Expressions in Urinary Bladder and Voided Cystometric Fluid

The microtiter plates (Phoenix Pharmaceuticals; Millipore Corporation) were coated with mouse anti-rat CGRP, anti-rat Sub P, or anti-rabbit-NT antibody. Sample and standard solutions were run in duplicate. Horseradish peroxidase- (HRP-) streptavidin or HRP-conjugated goat anti-rabbit IgG and LumiGLO were used to detect the antibody complex. Tetramethylbenzidine or LumiGLO was the substrate, and the enzyme activity or luminescence was measured. The CRGP standard provided with this protocol generated a standard curve from 0 to 100 ng/mL (*R* ^2^ = 0.998, *P* ≤ 0.0001) for bladder samples [30]. The Sub P standard provided with this protocol generated a standard curve from 0 to 25 ng/mL (*R* ^2^ = 0.998, *P* ≤ 0.0001) for bladder samples [30]. The nitrated-BSA standard provided with this protocol generated a standard curve from 1.5 to 100 *μ*g/mL (*R* ^2^ = 0.997, *P* ≤ 0.0001) for bladder samples. The absorbance values of standards and samples were corrected by subtraction of the background value (absorbance due to nonspecific binding) [18, 30]. No samples were diluted and all samples had absorbance values that fell onto the linear portion of the standard curve. Curve fitting of standards and evaluation of protein content of samples were performed using a least-squares fit.

### 2.6. Measurement of Cellular Oxidative Stress

The levels of cellular oxidative stress were determined by a spectrofluorimetric method, using the dichlorofluorescein DiOxyQ (DCFH-DiOxyQ) assay OxiSelect in vitro ROS/RNS Assay Kit (Green Fluorescence; Cell Biolabs, Inc., San Diego, CA, USA). The urinary bladder was removed from control (*n* = 6 each) and all experimental groups (*n* = 6 each) and rapidly homogenized in 50 mM Tris–Cl, pH 7.4. The homogenate was centrifuged at 2400 g for 15 min at 4°C and a low-speed supernatant fraction was used for assays. To determine levels of cellular oxidative stress, the supernatant from the urinary bladder homogenate was diluted (1 : 10) in 50 mM Tris–HCl (pH 7.4) and incubated with 10 *μ*L of 2′,7′-DCHF-DA (1 mM), at 37°C for 30 min. The DCHF-DA is enzymatically hydrolyzed by intracellular esterases to form nonfluorescent DCFH, which is then rapidly oxidized to form highly fluorescent 2′,7′-dichlorofluorescein (DCF) in the presence of cellular oxidative stress species. DCF fluorescence intensity is proportional to the amount of cellular oxidative stress species that is formed. The DCF fluorescence intensity emission was recorded at 520 nm (with 480 nm excitation) 30 min after the addition of DCHF-DA to the sample. The cellular oxidative species levels were expressed as fluorescence arbitrary units (FAU).

### 2.7. Collection and Measurement of Urinary ATP

#### 2.7.1. Sample Collections

For determination of ATP in urinary bladder, urinary bladder harvest and subsequent processing were performed as described above for ELISAs. For determination of ATP content in voided cystometric fluid, void volumes were collected following each micturition event in animal groups (control, CYP-treated groups, with and without Tempol) during conscious cystometry. For ATP measurements in voided cystometric fluid, 6–8 individual voids/animal were collected and immediately frozen on dry ice and stored at −80°C until use. Undiluted cystometric fluid samples were defrosted till 25°C and centrifuged at 3000 g at room temperature for 20 seconds to remove cellular debris and the supernatant was separated. Data from multiple voids were averaged, and the mean value was used for each animal.

#### 2.7.2. ATP Determination

A mixture of luciferin-luciferase was added according to the manufacturer instructions using the ATP Bioluminescence Assay Kit HS II (adenosine triphosphate (ATP) Bioluminescent Assay Kit, Sigma-Aldrich, St. Louis, MO, USA) as previously described [31]. ATP detection was evaluated using a multimode microplate reader (Synergy HT, BioTek Instruments Inc., Vermont, USA) controlled with Gen5 Data Analysis Software (BioTek). Sample bioluminescence was compared to that of standard amounts of ATP used in the same concentration range; standard ATP samples were prepared daily. All samples were run in duplicate. Cystometric void data were obtained from at least three voids/animal from control (*n* = 6 each) and all experimental groups (*n* = 6 each) and urinary bladder data were obtained from control (*n* = 6 each) and all experimental groups (*n* = 6 each). Vehicle for Tempol did not affect the ATP determinations. The ATP in cystometric fluid or ATP content in urinary bladder was calculated relative to the standard curve and expressed as nmol per total protein or nmol per mL of urine.

### 2.8. Conscious Cystometry and Effects of Tempol, a Superoxide Dismutase (SOD) Mimetic

Rats were anesthetized with isoflurane (3-4%), a lower midline abdominal incision was made, and polyethylene tubing (PE-50, Clay Adams, Parsippany, New Jersey) was inserted into the bladder dome and secured with nylon purse-string sutures (6-zero) [19, 27]. The end of the PE tubing was heat flared, but the catheter did not extend into the bladder body or neck and it was not associated with inflammation or altered cystometric function [19, 27]. The distal end of the tubing was sealed, tunneled subcutaneously, and externalized at the back of the neck out of reach of the animal [19, 27]. Abdominal and neck incisions were closed with nylon sutures (4-zero). Postoperative analgesics were given and animals were maintained for 72 to 96 hours after survival surgery to ensure recovery.

Tempol (4-hydroxy-2,2,6,6-tetramethylpiperidine-N-oxyl), a superoxide dismutase (SOD) mimetic, is a stable nitroxyl antioxidant. Previous studies have suggested that Tempol is protective in disorders involving ROS [32–35]. The effects of Tempol on urinary bladder function in CYP-treated (4 hr and 48 hr; *n* = 6 each) rats and control rats (*n* = 6 each) were assessed using conscious, open outlet, cystometry with continuous instillation of intravesical saline [19, 27]. Tempol was prepared daily with distilled water purified with a Millipore Milli-Q system and administered in the drinking water (1 mM) that was provided to rats* ad libitum*; untreated rats received water* ad libitum*. Covered bottles were used to minimize degradation by light. The addition of Tempol to the drinking water did not affect water consumption (data not shown). For CYP-treated rat groups (4 hr, 48 hr), Tempol was provided for approximately two weeks (13–15 days) while the intravesical tube was implanted on day 10, CYP or vehicle was injected on day 13, and rats were used in experiments on days 13–15. Due to the daily administration and route of delivery (oral) of the Tempol, these experiments were performed in different groups of control and CYP-treated rats treated with vehicle or Tempol. The concentration (1 mM) of Tempol and duration of treatment used in these studies were based upon previous studies [36, 37].

### 2.9. Exclusion Criteria

Rats were removed from the study when adverse events occurred that included 20% reduction in body weight postsurgery, a significant postoperative event, lethargy, pain, or distress not relieved by our IACUC-approved regimen of postoperative analgesics or hematuria in control rodents [19, 27]. In the present study, no rats were excluded from the study. In addition, behavioral movements such as grooming, standing, walking, and defecation rendered bladder pressure recordings during these events unusable.

### 2.10. Statistical Analyses

All values represent mean ± SEM. Cystometry data were compared using repeated measures ANOVA, where each animal served as its own control. Animals, processed and analyzed on the same day, were tested as a block in the ANOVA. When *F*-ratios exceeded the critical value (*P* ≤ 0.05), the Newman-Keuls or Dunnett's post hoc tests were used to compare group means. Data obtained from the ATP assays violated the assumptions of the ANOVA. Thus, these data were analyzed using a nonparametric analysis, the Mann-Whitney Rank Sum test. When *F* ratios exceeded the critical value (*P* ≤ 0.05), the Newman-Keuls post hoc test was used to compare the experimental means. *P* ≤ 0.05 (two-tailed) values were considered statistically significant.

## 3. Results

### 3.1. ROS/RNS Expression in Urinary Bladder with CYP-Induced Cystitis and the Effects of Tempol

**Figure 1:**
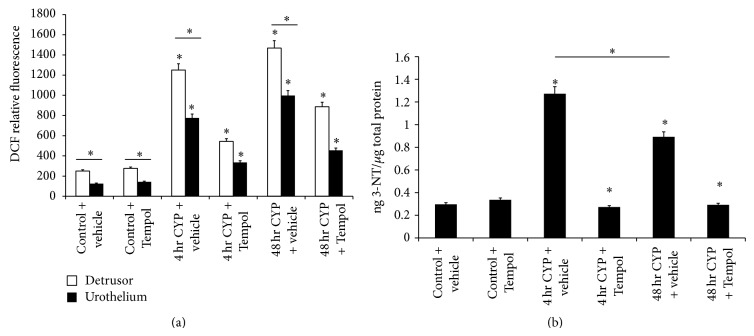
Cyclophosphamide- (CYP-) induced cystitis increases reactive oxygen species (ROS)/reactive nitrogen species (RNS) and 3-nitrotyrosine (3-NT) in the urinary bladder and the antioxidant, Tempol, reduces expression. (a) 4 hr and 48 hr CYP-induced cystitis significantly (*P* ≤ 0.01) increased ROS/RNS expression in urothelium and detrusor that was significantly (*P* ≤ 0.01) reduced with Tempol. Basal expression and CYP-induced ROS/RNS expression were significantly (*P* ≤ 0.01) greater in detrusor compared to urothelium. Tempol was without effect on ROS/RNS expression in urothelium and detrusor from control (no CYP) rats. (b) 4 hr and 48 hr CYP-induced cystitis significantly (*P* ≤ 0.01) increased ROS/RNS expression in urinary bladder that was significantly (*P* ≤ 0.01) reduced with Tempol. Tempol was without effect on 3-NT expression in urinary bladder from control (no CYP) rats. ^*^ *P* ≤ 0.01. *n* = 6 for control and treatment groups.

We determined the levels of reactive oxygen species (ROS) and reactive nitrogen species (RNS) in the urinary bladder following CYP treatment (4 hr, 48 hr) and in the presence of the antioxidant, Tempol. 4 hr and 48 hr CYP-induced cystitis significantly (*P* ≤ 0.01) increased the total free radical presence in the detrusor and urothelium that was significantly reduced (*P* ≤ 0.01) by Tempol (1 mM) delivered in the drinking water (Figure 1(a)). With both 4 hr and 48 hr CYP-induced cystitis, the increase in total free radical presence was significantly (*P* ≤ 0.01) greater in the detrusor compared to the urothelium (Figure 1(a)). Basal expression of total free radical presence in the detrusor was significantly (*P* ≤ 0.01) greater in the detrusor compared to the urothelium (Figure 1(a)).

### 3.2. 3-Nitrotyrosine (3-NT) Expression in Urinary Bladder with CYP-Induced Cystitis and the Effects of Tempol

3-NT, a product of tyrosine nitration mediated by RNS such as peroxynitrite anion and nitrogen dioxide, is considered a marker of NO-dependent, RNS-induced nitrative stress [38]. 4 hr and 48 hr CYP-induced cystitis significantly (*P* ≤ 0.01) increased 3-NT expression in the urinary bladder that was significantly reduced (*P* ≤ 0.01) by Tempol (1 mM) delivered in the drinking water (Figure 1(b)). 3-NT expression in the urinary bladder was significantly (*P* ≤ 0.01) greater following 4 hr CYP-induced cystitis compared to 48 hr CYP-induced cystitis (Figure 1(b)).

### 3.3. Substance P (Sub P) and Calcitonin Gene-Related Peptide (CGRP) Expression in Urinary Bladder and Cystometric Fluid with CYP-Induced Cystitis and the Effects of Tempol

**Figure 2:**
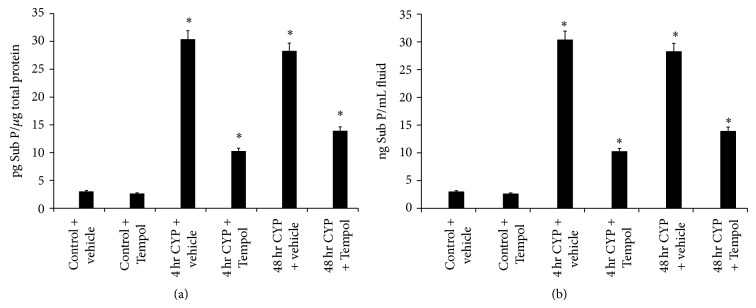
Cyclophosphamide- (CYP-) induced cystitis increases substance P (Sub P) in the urinary bladder and cystometric fluid and the antioxidant, Tempol, reduces expression. (a) 4 hr and 48 hr CYP-induced cystitis significantly (*P* ≤ 0.01) increased Sub P expression in urinary bladder that was significantly reduced with Tempol. Tempol was without effect on Sub P expression in urinary bladder from control (no CYP) rats. (b) 4 hr and 48 hr CYP-induced cystitis significantly (*P* ≤ 0.01) increased Sub P expression in cystometric fluid that was significantly (*P* ≤ 0.01) reduced with Tempol. Tempol was without effect on Sub P expression in cystometric fluid from control (no CYP) rats. ^*^ *P* ≤ 0.01. *n* = 6 for control and treatment groups.

**Figure 3:**
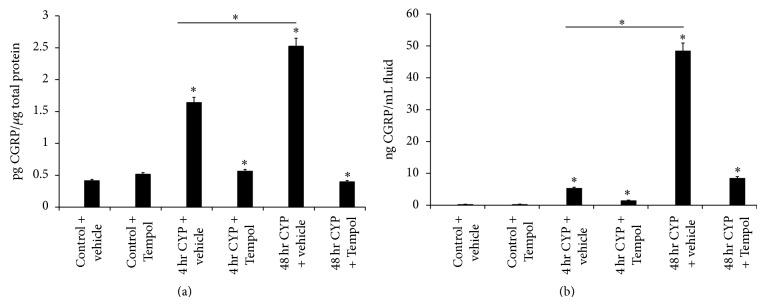
Cyclophosphamide- (CYP-) induced cystitis increases calcitonin gene-related peptide (CGRP) in the urinary bladder and cystometric fluid and the antioxidant, Tempol, reduces expression. (a) 4 hr and 48 hr CYP-induced cystitis significantly (*P* ≤ 0.01) increased CGRP expression in urinary bladder that was significantly (*P* ≤ 0.01) reduced with Tempol. Tempol was without effect on CGRP expression in urinary bladder from control (no CYP) rats. (b) 4 hr and 48 hr CYP-induced cystitis significantly (*P* ≤ 0.01) increased CGRP expression in cystometric fluid that was significantly (*P* ≤ 0.01) reduced with Tempol. ^*^ *P* ≤ 0.01. *n* = 6 for control and treatment groups.

The neuropeptides, Sub P and CGRP, are known modulators of inflammation and may contribute to the pathogenesis of many diseases including migraine, asthma, and urinary bladder inflammation [4, 20, 39, 40]. 4 hr and 48 hr CYP-induced cystitis significantly (*P* ≤ 0.01) increased Sub P and CGRP expression in the urinary bladder (Figures 2(a) and 3(a)) and cystometric fluid (Figures 2(b) and 3(b)) that was significantly reduced (*P* ≤ 0.01) by Tempol (1 mM) delivered in the drinking water (Figures 2(a), 2(b), 3(a) and 3(b)). Sub P expression in the urinary bladder was similar following 4 hr and 48 hr CYP-induced cystitis in the urinary bladder and cystometric fluid (Figures 2(a) and 2(b)). In contrast, 48 hr CYP-induced cystitis resulted in significantly (*P* ≤ 0.01) greater CGRP expression in the urinary bladder and cystometric fluid compared to the 4 hr time point (Figures 3(a) and 3(b)).

### 3.4. Adenosine Triphosphate (ATP) Expression in Urinary Bladder and Cystometric Fluid with CYP-Induced Cystitis and the Effects of Tempol

**Figure 4:**
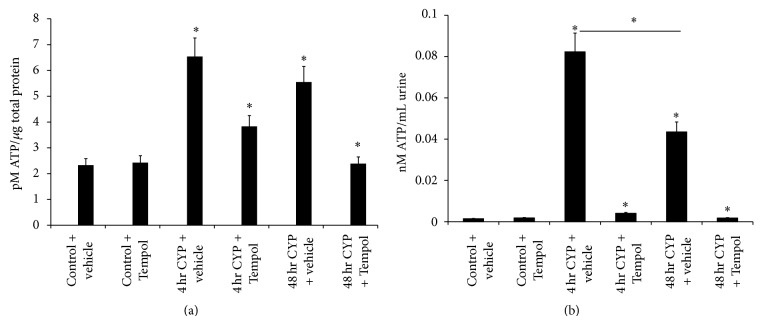
Cyclophosphamide- (CYP-) induced cystitis increases adenosine triphosphate (ATP) in the urinary bladder and cystometric fluid and the antioxidant, Tempol, reduces expression. (a) 4 hr and 48 hr CYP-induced cystitis significantly (*P* ≤ 0.01) increased ATP expression in urinary bladder that was significantly (*P* ≤ 0.01) reduced with Tempol. Tempol was without effect on ATP expression in urinary bladder from control (no CYP) rats. (b) 4 hr and 48 hr CYP-induced cystitis significantly (*P* ≤ 0.01) increased ATP expression in cystometric fluid that was significantly (*P* ≤ 0.01) reduced with Tempol. Tempol was without effect on ATP expression in cystometric fluid from control (no CYP) rats. ^*^ *P* ≤ 0.01. *n* = 6 for control and treatment groups.

Numerous studies have described a role(s) for ATP in urinary bladder dysfunction, pain, and altered ATP release mechanisms in animal models and clinical studies of BPS/IC [8]. 4 hr and 48 hr CYP-induced cystitis significantly (*P* ≤ 0.01) increased ATP expression in the urinary bladder and cystometric fluid that was significantly reduced (*P* ≤ 0.01) by Tempol (1 mM) delivered in the drinking water (Figures 4(a) and 4(b)). ATP expression in the urinary bladder was similar following 4 hr and 48 hr CYP-induced cystitis (Figure 4(a)). In contrast, 4 hr CYP-induced cystitis resulted in significantly (*P* ≤ 0.01) greater ATP expression in the cystometric fluid compared to the 48 hr time point (Figure 4(b)).

### 3.5. Effect of an Antioxidant, Tempol, on Bladder Function

**Figure 5:**
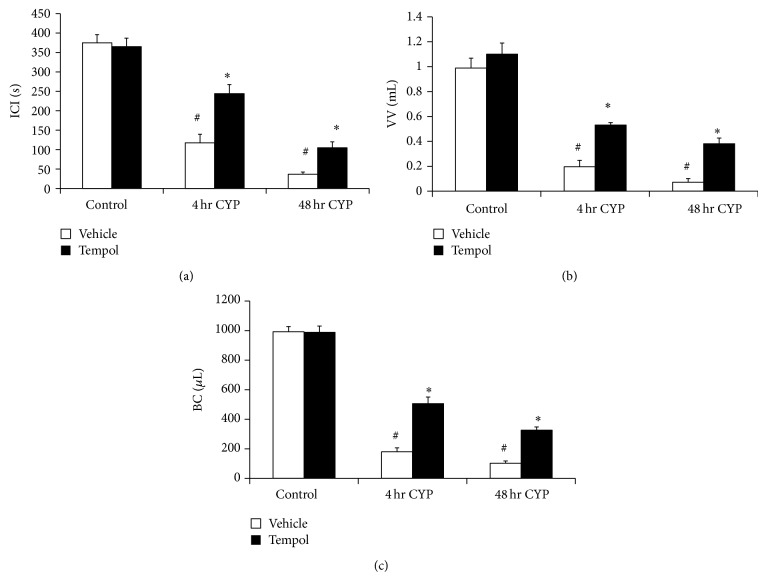
Summary histograms of the effects of Tempol (1 mM) on intercontraction interval (ICI; s), bladder capacity (BC; *μ*L), and void volume (VV; mL) in CYP-treated (4 hr; 48 hr) rats. Tempol in the drinking water significantly (*P* ≤ 0.01) increased ICI (a), VV (b), and BC (c) in CYP-treated (4 hr and 48 hr) rats. Tempol was without effect in control (no CYP treatment) rats. ^#^ *P* ≤ 0.01 compared to control + vehicle (between-group difference); ^*^ *P* ≤ 0.01 compared to 4 hr or 48 hr CYP + vehicle (within-group difference). Sample sizes are *n* = 6 in control and treatment groups.

**Figure 6:**
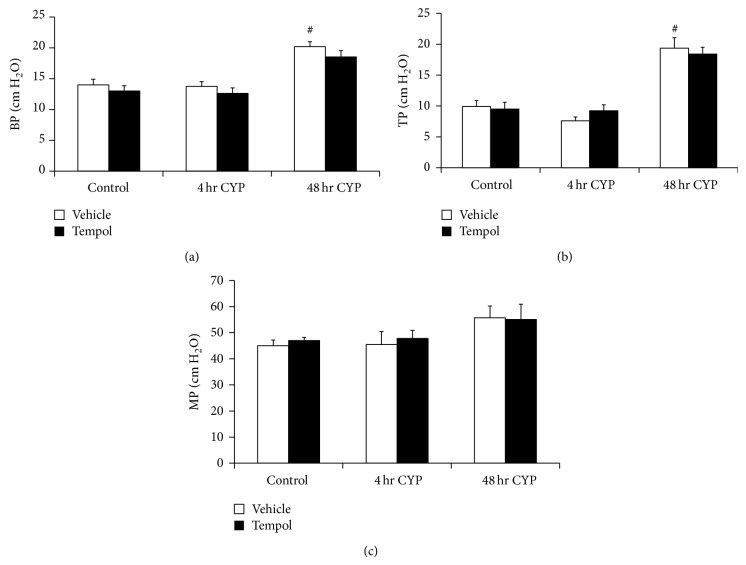
Tempol in the drinking water was without effect on baseline pressure ((a); BP), threshold pressure ((b); TP), or peak micturition pressure ((c); MP; cm H_2_O) in control or CYP-treated (4 hr; 48 hr) rats. Sample sizes are *n* = 6 in control and treatment groups. ^#^ *P* ≤ 0.01 compared to control + vehicle (between-group difference).

**Figure 7:**
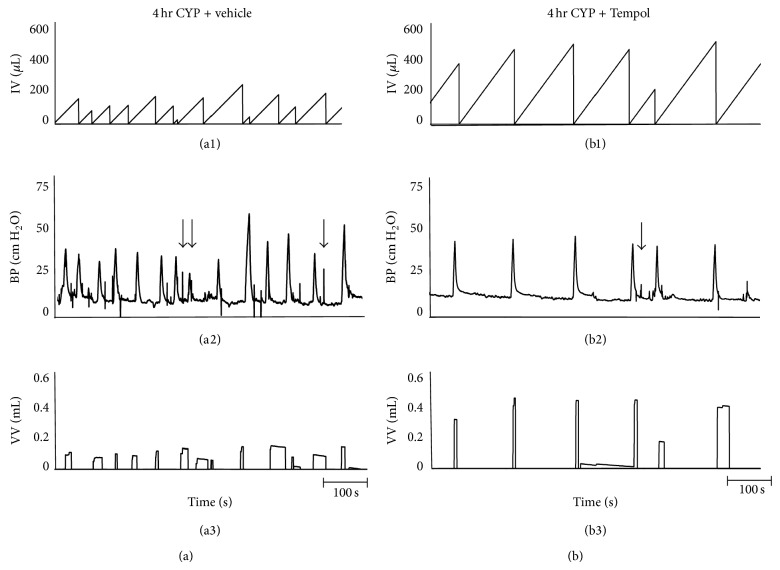
Representative cystometrogram recordings using continuous intravesical infusion of saline in conscious rats with an open outlet from a CYP-treated (4 hr) rat with vehicle ((a1)–(a3)) and a CYP-treated (4 hr) rat with Tempol (1 mM; (b1)–(b3)). ((a), (b)) Bladder function in a CYP-treated (4 hr) rat without Tempol (vehicle only; (a1)–(a3)) and in a CYP-treated (4 hr) rat with Tempol (1 mM in the drinking water; (b1)–(b3)) during continuous intravesical infusion of saline. Bladder function recordings in (a) and (b) are from different rats. Infused volume (IF, *μ*L; (a1), (b1)), bladder pressure (BP, cm H_2_O; (a2), (b2)), and void volume (VV, mL; (a3), (b3)) with vehicle ((a1)–(a3)) and with Tempol treatment ((b1)–(b3)) are shown. Arrows ((a2), (b2)) indicate examples of nonvoiding contractions that were significantly (*P* ≤ 0.01) reduced in number and amplitude in CYP-treated (4 hr) rats with Tempol.

**Figure 8:**
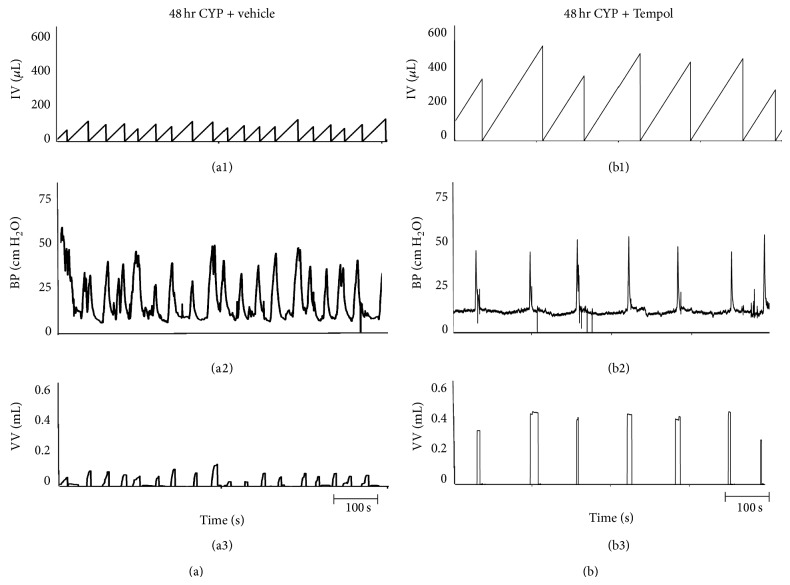
Representative cystometrogram recordings using continuous intravesical infusion of saline in conscious rats with an open outlet from a CYP-treated (48 hr) rat with vehicle ((a1)–(a3)) and a CYP-treated (48 hr) rat with Tempol (1 mM; (b1)–(b3)). ((a), (b)) Bladder function in a CYP-treated (48 hr) rat without Tempol (vehicle only; (a1)–(a3)) and in a CYP-treated (48 hr) rat with Tempol (1 mM in the drinking water; (b1)–(b3)) during continuous intravesical infusion of saline. Bladder function recordings in (a) and (b) are from different rats. Infused volume (IF, *μ*L; (a1), (b1)), bladder pressure (BP, cm H_2_O; (a2), (b2)), and void volume (VV, mL; (a3), (b3)) with vehicle ((a1)–(a3)) and with Tempol treatment ((b1)–(b3)) are shown.

Conscious, open outlet cystometry with continuous intravesical infusion of saline was performed in separate groups (*n* = 6 each) of control and CYP-treated (48 h) rats with or without Tempol (vehicle only) in the drinking water to determine bladder function (Figures 5–8).

#### 3.5.1. Control (No CYP Treatment)

Tempol (1 mM) treatment had no effects on ICI, BC, or VV in control rats (no CYP treatment) compared with control rats (no CYP treatment) treated with vehicle (Figure 5). There were no changes in BP, TP, or peak MP with Tempol treatment compared to control rats (no CYP treatment) treated with vehicle (Figure 6). Residual volume in control rats with or without Tempol (vehicle) treatment was minimal (≤10 *μ*L).

#### 3.5.2. CYP Treatment

As previously demonstrated [22–24] and confirmed here, CYP treatment (4 hr and 48 hr) increased void frequency and decreased BC, ICI, and VV compared with control rats (no CYP treatment) (Figures 5–8). Additionally, 48 hr CYP-induced cystitis significantly (*P* ≤ 0.01) increased BP and TP (Figure 6). Tempol in the drinking water (1 mM) of CYP-treated rats (4 hr and 48 hr) significantly (*P* ≤ 0.01) increased the ICI (i.e., decreased voiding frequency) (Figure 5(a); 2.0–2.9-fold), increased BC (Figure 5(c); 2.8–3.1-fold), and increased VV (Figure 5(b); 2.9–5.4-fold) compared to rats treated with CYP (4 hr and 48 hr) receiving vehicle (Figures 7 and 8). Tempol treatment of CYP-treated rats (4 hr and 48 hr) increased BC to 70% of control rats (no CYP treatment) (Figures 5(c), 7, and 8). Effects of Tempol on bladder function in CYP-treated rats persisted for at least 2 hr. Residual volume in CYP-treated (4 hr and 48 hr) rats with or without Tempol treatment was minimal and similar to that observed in control (no CYP treatment) (≤10 *μ*L). CYP-treated rats (4 hr and 48 hr) treated with or without Tempol (vehicle) exhibited no differences in BP, TP, or peak MP (Figures 6(a)–6(c)). Tempol also significantly (*P* ≤ 0.01) reduced the number (2.4 ± 0.4/micturition cycle versus 0.6 ± 0.2/micturition cycle) and amplitude (11.2 ± 1.3 cm H_2_O versus 7.5 ± 0.5 cm H_2_O) of NVCs (increases in bladder pressure during the filling phase without the release of fluid) in 4 hr CYP-treated rats (Figure 7). The effects of Tempol on NVCs in the 48 hr CYP-treated group were not determined due to the dramatically increased voiding frequency that made the presence of NVCs difficult to determine.

## 4. Discussion

The present studies demonstrate several novel findings with respect to the induction and reduction of oxidative stress with the antioxidant, Tempol, following cyclophosphamide- (CYP-) induced cystitis. CYP-induced cystitis (4 hr and 48 hr) increased ROS/RNS and 3-NT expression in the urinary bladder. In addition, CYP-induced cystitis increased expression of neuropeptides, CGRP, and Sub P, in the urinary bladder as well as cystometric fluid collected during conscious cystometry. CYP-induced cystitis also increased ATP in the urinary bladder and cystometric fluid. Providing rats with the antioxidant, Tempol, in the drinking water prior to and during the induction of CYP-induced cystitis significantly reduced the expression of ROS/RNS, CGRP, Sub P, and ATP in urinary bladder and cystometric fluid. Further, Tempol decreased voiding frequency and increased the intercontraction interval and bladder capacity without effects on urinary bladder pressures (baseline, threshold, and peak) in rats with CYP-induced cystitis. Tempol did not alter bladder function in control (no CYP treatment) rats. These studies demonstrate that CYP-induced cystitis is associated with oxidative stress in the urinary tract and that use of the antioxidant, Tempol, reduces oxidative stress and improves urinary bladder function.

Molecular oxygen reduction results in the production of several reactive intermediates that must be actively scavenged [41]. Under conditions of inflammation, there may be insufficient scavenging of reactive intermediates that can lead to oxidative stress and damage to cellular structure and function. Recent studies suggest that the generation of these reactive intermediates contributes to the pathogenesis of urinary bladder dysfunction with CYP administration [42–44]. The contribution of oxidative stress in bladder inflammation following CYP is further supported by the attenuation of tissue damage following the administration of agents with antioxidant properties, like taurine [42], flavonoid [43], beta-carotene, and others [44]. Our studies using the CYP model of urinary bladder inflammation at two time points (4 hr and 48 hr) confirm the expression of reactive intermediates including ROS/RNS and 3-NT in the urinary bladder. Expression of ROS/RNS following CYP-induced cystitis was significantly greater in the detrusor smooth muscle compared to the urothelium but both tissues exhibited increased ROS/RNS compared to control (no CYP treatment) urinary bladder. 3-NT expression has been previously demonstrated in lower urinary tract tissues following injury or inflammation. Partial outlet obstruction in rabbit is associated with increased NT in mucosa [45] that is correlated with progressive decrease in contractility of detrusor smooth muscle [46]. Previous studies demonstrated iNOS and NT immunoreactivity in the urothelium and inflammatory infiltrates in the lamina propria of individuals with BPS/IC with a Hunner's lesion [47]. Consistent with this clinical study, the present study demonstrates increased 3-NT in the urinary bladder following CYP-induced cystitis (4 hr and 48 hr) in a rat model. The amino acid tyrosine is particularly susceptible to nitration and the formation of 3-NT may represent a biomarker for the generation of reactive nitrogen intermediates in vivo [47]. In addition to being a biomarker, 3-NT could also have a detrimental impact on cell function and viability by inhibiting protein phosphorylation by tyrosine kinases and interfering with the signal transduction mechanism [48]. In addition, in vitro nitration of a specific tyrosine residue inactivates manganese superoxide dismutase [49] that may lead to a greater concentration of ROS/RNS perpetuating tissue damage and altered function.

As a major sensory component of the urinary bladder, the urothelium is able to respond to various extracellular stimuli by releasing neuroactive factors like ATP, acetylcholine, and nitric oxide [8]. The sustained release of these factors, such as what may occur with inflammation, may underlie the development of urinary bladder dysfunction and lower urinary tract symptoms. The role of oxidative stress with ATP release in epithelial cells, however, is not well defined. In endothelial cells, oxidative stress has been demonstrated to mediate the direct release of ATP and inhibit the catabolism of extracellular ATP [50, 51]. Further, purinergic receptor activation has been associated with increased cellular production and release of multiple inflammatory mediators, including superoxide anion, nitric oxide, and other ROS. Purinergic receptor activation induces ROS generation in numerous cell types resulting in a variety of downstream effects including transcription factor activation [13], proinflammatory cytokine release [14, 15], and cell death [16]. Our studies determined the contribution of ROS/RNS to extracellular ATP expression with CYP-induced cystitis and Tempol. CYP-induced cystitis increased ATP expression in the urinary bladder and cystometric fluid that was significantly reduced by Tempol administration. It is not known from the present studies whether antioxidant treatment directly inhibits the release of ATP through purinergic receptor blockade. Additional studies in CYP-treated rats involving assessment of oxidative stress in the presence of purinergic receptor (P2) blockade as well as the effects of P2 activation on oxidative stress in controls should be considered.

Tempol is a membrane-permeable, redox-cycling agent that scavenges superoxide anions and decreases the formation of hydroxyl radicals [35]. The protective effects of Tempol to tissues with inflammation and oxidative damage have been widely established. For example, Tempol has been shown to decrease NF-kappaB activation with acute inflammation [52], decrease neutrophil infiltration and PARP activation with periodontitis [53], and decrease cytokine release stimulated by an inflammatory soup [54]. In animal studies, purinergic neuromuscular transmission and propulsive motility were significantly restored in the inflamed colon treated with the free radical scavenger, Tempol [55]. Furthermore, intrathecal Tempol administration has been demonstrated to decrease thermal and mechanical hypersensitivity with neuropathic pain [56]. Due to the central and peripheral anti-inflammatory properties of Tempol, our studies determined the role of oxidative stress on bladder function with CYP-induced cystitis and Tempol administration. Pretreatment with Tempol and continued treatment with Tempol during the progression of CYP-induced cystitis significantly improved urinary bladder function. In CYP-treated (4 hr and 48 hr) rats, Tempol in the drinking water increased the intercontraction interval and bladder capacity and reduced urinary frequency. The presence and amplitude of NVCs during the filling phase of the urinary bladder were also reduced in 4 hr CYP-treated rats given Tempol. Given the demonstration of Tempol decreased mechanical hypersensitivity from neuropathic pain [56], future studies should examine the effects of Tempol administration on somatic (i.e., pelvic and hindpaw) sensitivity in CYP-treated rats.

Previous studies from our laboratory have demonstrated roles for neuropeptides, including pituitary adenylate cyclase-activating polypeptide (PACAP), CGRP, and Sub P in CYP-induced bladder dysfunction [20, 21]. Increased expression of PACAP, CGRP, and Sub P was demonstrated in the urinary bladder, lumbosacral spinal cord, and dorsal root ganglia of CYP-treated rats and pharmacological blockade of the PACAP specific PAC1 receptor improved urinary bladder function in CYP-treated rats [20, 21]. The present studies confirm increased expression of Sub P and CGRP in the urinary bladder and cystometric fluid with CYP-induced cystitis (4 hr and 48 hr). Tempol treatment significantly reduced Sub P and CGRP expression in the urinary bladder and cystometric fluid. Previous studies demonstrated that Sub P via neurokinin (NK) receptor facilitates bladder afferent signaling and ROS formation in bladder in association with neurogenic inflammation [57]. Increased Sub P release increased ROS in the bladder via increased mast cell degranulation, intercellular adhesion molecule expression, and leukocyte adhesion. Future studies can determine the involvement of Sub P/NK receptor signaling with these sources of ROS in the inflamed bladder following CYP-induced cystitis. The neuropeptides, PACAP27, PACAP38 and VIP, evoked ATP release from rat urothelial cell cultures that was significantly blocked by the PAC1 receptor selective antagonist, M65 [31]. The present studies suggest two possibilities by which CYP-induced cystitis increases ATP expression in the urinary bladder and cystometric fluid: (1) neuropeptide (Sub P and CGRP) evoked release of ATP and (2) ROS evoked ATP release. Current research is consistent with the suggestion that neuropeptide and ROS signaling are regulators of bladder physiology at the level of the urinary bladder and possibly the urothelium [31]. The present studies demonstrate that use of the antioxidant, Tempol, not only reduces the presence of oxidative stress markers in the urinary tract but also reduces modulators/mediators of inflammation including neuropeptides (CGRP, Sub P) also known to contribute to micturition reflex plasticity and dysfunction with CYP-induced cystitis. In the context of CYP-induced cystitis, Tempol treatment may be more beneficial given its broader impact on oxidative stress markers and other modulators of urinary bladder inflammation while also improving urinary bladder function.

The present studies demonstrate oxidative stress in the urinary tract following CYP-induced cystitis and improvement in urinary bladder function and markers of oxidative stress with antioxidant treatment; however, there are additional issues to be considered. Future studies may include (1) determining the effects of Tempol after the induction of CYP-induced cystitis, rather than as a pretreatment, on urinary bladder function; (2) determining if Tempol treatment can reduce somatic sensitivity in the CYP model of urinary bladder inflammation with referred, somatic hypersensitivity; (3) determining the effects of Tempol in a more chronic model of CYP-induced urinary bladder inflammation that we have used extensively [25, 26]. The present studies demonstrate that CYP-induced cystitis results in oxidative stress in the urinary tract and that the antioxidant, Tempol, ameliorates CYP-induced bladder dysfunction. These studies suggest that pharmacological interventions directed at oxidative stress mediators/markers may be a promising strategy to address inflammation of the urinary tract and target organ dysfunction.
